# Chemical aminoacylation of tRNAs with fluorinated amino acids for in vitro protein mutagenesis

**DOI:** 10.3762/bjoc.6.40

**Published:** 2010-04-20

**Authors:** Shijie Ye, Allison Ann Berger, Dominique Petzold, Oliver Reimann, Benjamin Matt, Beate Koksch

**Affiliations:** 1 Department of Biology, Chemistry and Pharmacy, Freie Universität Berlin, Institute of Chemistry and Biochemistry – Organic Chemistry, Takustrasse 3, 14195 Berlin, Germany; Tel.: 0049-30-83855344; Fax: 0049-30-83855644

**Keywords:** chemical aminoacylation, DfeGly, fluorinated amino acids, site-specific protein mutagenesis, TfeGly, TfmAla

## Abstract

This article describes the chemical aminoacylation of the yeast phenylalanine suppressor tRNA with a series of amino acids bearing fluorinated side chains via the hybrid dinucleotide pdCpA and ligation to the corresponding truncated tRNA species. Aminoacyl-tRNAs can be used to synthesize biologically relevant proteins which contain fluorinated amino acids at specific sites by means of a cell-free translation system. Such engineered proteins are expected to contribute to our understanding of discrete fluorines’ interaction with canonical amino acids in a native protein environment and to enable the design of fluorinated proteins with arbitrary desired properties.

## Introduction

**Figure 1:**
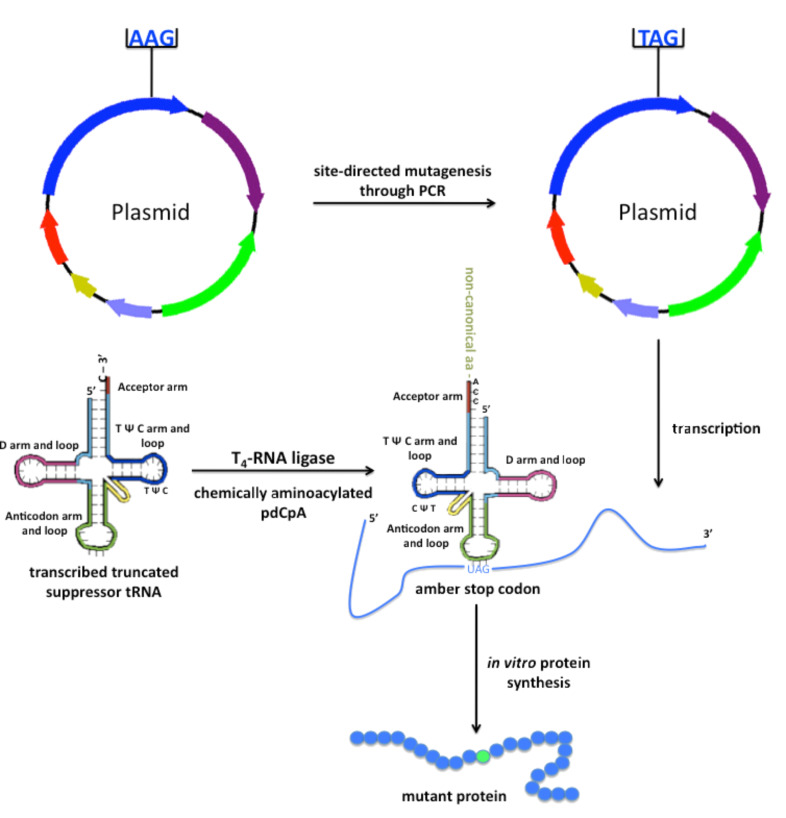
General strategy of using a chemically aminoacylated suppressor tRNA and an in vitro translation system to produce proteins which contain non-canonical amino acids (based on Noren et al. [1]). An amber stop codon, TAG, is introduced site-specifically into the protein-encoding gene, replacing an amino acid-encoding codon. The hybrid dinucleotide pdCpA is chemically aminoacylated with the non-canonical amino acid of interest and ligated to the appropriately truncated suppressor tRNA (tRNA-CA). Non-natural mutant proteins are produced by means of an in vitro protein synthesis system.

Over the past two decades, the interest in engineering proteins containing site-specific synthetic amino acids with novel functionalities has grown considerably. The utility of chemically aminoacylated suppressor transfer RNAs (tRNAs) combined with cell-free translation systems in producing proteins that contain non-canonical amino acids was reported independently from each other by Schultz and Chamberlin [1–2]. Their methodology is based on the following observations: 1) the central intermediate molecule in protein translation, the aminoacyl-tRNA (aa-tRNA) produced in the cell by specific tRNA synthetases (aaRSs) can be semi-synthesized; 2) a nonsense codon TAG can replace an amino acid-encoding codon at a desired position and can be recognized by the corresponding mutated orthogonal suppressor tRNA during the translation (Figure 1).

The key intermediates in this methodology are the suppressor aminoacyl-tRNAs. Due to the great number of reactive groups in the tRNA molecule, direct chemical acylation is not possible. Hecht and co-workers developed a procedure in which *N*^α^-protected amino acids were used to chemically aminoacylate the dinucleotide pCpA. Subsequent enzymatic ligation to truncated tRNAs (without the 3′-terminal CA dinucleotide) yielded the desired AA-tRNAs [3]. More recently, this approach was optimized and the chemistry simplified by Schultz and co-workers. By using the hybrid dinucleotide pdCpA and activation of the amino acid as the cyanomethyl ester, selective coupling to the 2′- or 3′-hydroxyl group of the terminal adenosine was possible [4–5]. Since the *N*^α^-aminoacyl moiety is not stable under the ligation conditions, the *N*^α^-amino group of amino acid was either protected beforehand as the 6-nitroverastryloxycarbonyl (NVOC) derivative, a moiety which can be removed photochemically after the ligation, or left unprotected. However, in both cases low yields resulted. Lately, Hecht and co-workers reported the use of the *N*-(4-pentenoyl) protecting group as suitable for preparing a variety of misacylated tRNAs [6–9]. Removal of this group is achieved under mild chemical conditions by treatment with iodine solution; it has also been used in the preparation of caged proteins.

Fluorine is the most electronegative element and has a van der Waals radius of 1.47 Å [10]. Thus, substitution of a C–H bond with a C–F bond dramatically changes the electronic properties of the given molecule but exerts only a minor steric effect [11]. Due to the unique properties of the fluorine atom, the incorporation of amino acids which contain fluorinated side chains into peptides and proteins is becoming increasingly popular for the rational design of biopolymers and materials with novel biological properties. For example, certain fluorinated analogues of hydrophobic amino acids have been incorporated into the hydrophobic core of peptides, oligomers and proteins, leading to a significant increase in the thermal stability of the structure [12–15]. The introduction of fluoroalkyl groups into proteins can also enhance the hydrophobicity of the molecule, enabling better diffusion across the membranes [16]. Koksch and co-workers have developed a model peptide system based on the coiled-coil folding motif. They used it to show that the impact of fluorine substitution on structure and stability is strongly dependent on the position and the number of fluorine atoms within the peptide chain [17–19]. Finally, due to the high NMR sensitivity of fluorine, the incorporation of fluorinated amino acid analogues into proteins provides the opportunity for probing the structure and dynamics that play a role in protein–protein and protein–ligand interaction, and metabolic processes [20–21].

**Figure 2:**
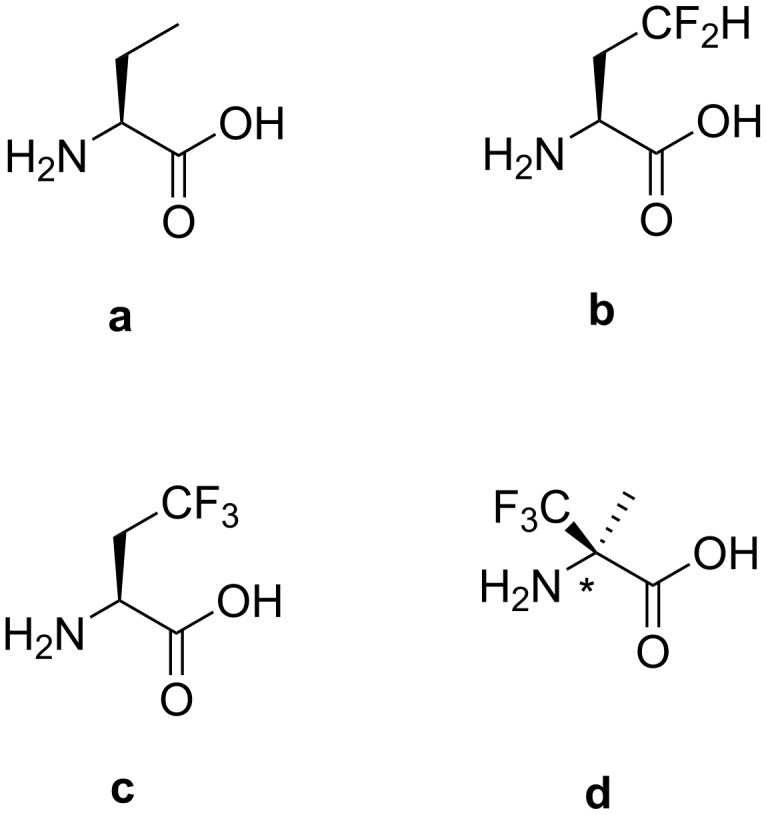
Structures of non-canonical amino acids. **a.** (*S*)-ethylglycine, **b.** (*S*)-2-amino-4,4-difluorobutanoic acid, **c.** (*S*)-2-amino-4,4,4-trifluorobutanoic acid, **d.** (*RS*)-2-amino-2-methyl-3,3,3-trifluoropropanoic acid.

We report here the chemical and enzymatic aminoacylation of the yeast phenylalanine suppressor tRNA with a series of fluoroalkylated amino acids for site-specific protein mutagenesis (Figure 2). (*RS*)-2-amino-2-methyl-3,3,3-trifluoropropanoic acid (α-(Tfm)Ala) [22], (*S*)-ethylglycine (Abu) and two of its fluorinated analogues, (*S*)-2-amino-4,4-difluorobutanoic acid (DfeGly) [23] and (*S*)-2-amino-4,4,4-trifluorobutanoic acid (TfeGly) [24], were synthesized in the appropriate protected activated form and used to chemically aminoacylate tRNA^Phe^_CUA_ by means of the hybrid dinucleotide pdCpA and enzymatic ligation.

## Results and Discussion

### Syntheses of *N*-(4-pentenoyl) amino acid cyanomethyl esters

**Scheme 1:**
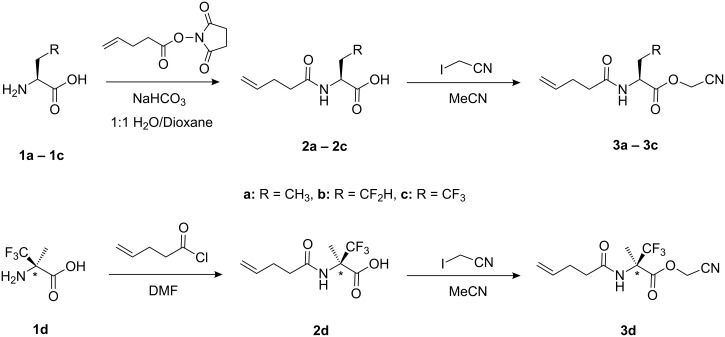
General scheme for the synthesis of an *N*-(4-pentenoyl) amino acid cyanomethyl ester.

The aminoacylation of a suppressor tRNA is the first step to incorporate non-canonical amino acids into proteins. Several strategies have been developed to accomplish this [4–7,25–27]. We chose a combination of chemical and enzymatic aminoacylation which relies on the hybrid dinucleotide pdCpA and the T4-RNA ligase-mediated coupling to it to give a truncated suppressor tRNA. The *N*^α^-amino groups of the amino acids were protected with the 4-pentenoyl group and the amino acids were activated as their corresponding cyanomethyl esters (Scheme 1).

The *N*-(4-pentenoyl) protection of Abu and its fluorinated analogues DfeGly and TfeGly, and the preparation of their cyanomethyl esters were performed as described by Hecht and co-workers [7,9]. In the first step, the amino acid was treated with *N*-(4-pentenoyloxy)succinimide and in the second step treatment with iodoacetonitrile gave the desired compound in yields ranging from 59 to 81%. Due to the strong electron-withdrawing character of the C–F bond, the CF_3_ substituent in the α-position in α-(Tfm)Ala influences considerably the reactivity of both the amino and carboxylic groups; there is also a steric effect in this case. The amino group of α-(Tfm)Ala is generally protected by treatment with highly reactive mixed anhydrides or acid chlorides [28]. Thus, the *N*-(4-pentenoyl)-α-(Tfm)Ala was synthesized by means of 4-pentenoyl chloride. We investigated the reaction both in pyridine and DMF as the solvent, in the case of DMF, 4-dimethylaminopyridine (DMAP) was added as base. Although, pyridine also behaves as a base (p*K*_a_ 5.21), higher yields were achieved with DMAP in DMF. The synthesis of *N*-(4-pentenoyl)-TfmAla cyanomethyl ester was achieved in an overall yield of 22%. The syntheses of *N*-(4-pentenoyl) amino acid cyanomethyl esters are summarized in Table 1.

**Table 1:** Syntheses of *N*-(4-pentenoyl) amino acid cyanomethyl esters.

	Yield of protection and activation (%)	**^19^**F-NMR: δ (ppm)^a^	Mass (M+H)^+^ (*m/z*) (calculated)

Abu (**3a**)	81	—	225.1263 (224.1161)
DfeGly (**3b**)	59	−116.46 (tdd, 1F, *J* = 320.0 Hz, *J* = 58.6 Hz, *J* = 17.1 Hz), −115.70 (tdd, 1F, *J* = 320.0 Hz, *J* = 58.6 Hz, *J* = 17.1 Hz)	261.1045 (260.0972)
TfeGly (**3c**)	75	−62.98 (t, 3F, *J* = 9.8 Hz)	279.0929 (278.0878)
TfmAla (**3d**)	22	−76.227 (s, 3F)	279.0929 (278.0878)

^a^s: singlet, t: triplet (See Supporting Information File 1).

### Syntheses of 2′(3′)-*O*-[*N*-(4-pentenoyl)aminoacyl]-tRNAs and bis-2′,3′-*O*-[*N*-(4-pentenoyl)aminoacyl]-tRNAs

**Scheme 2:**
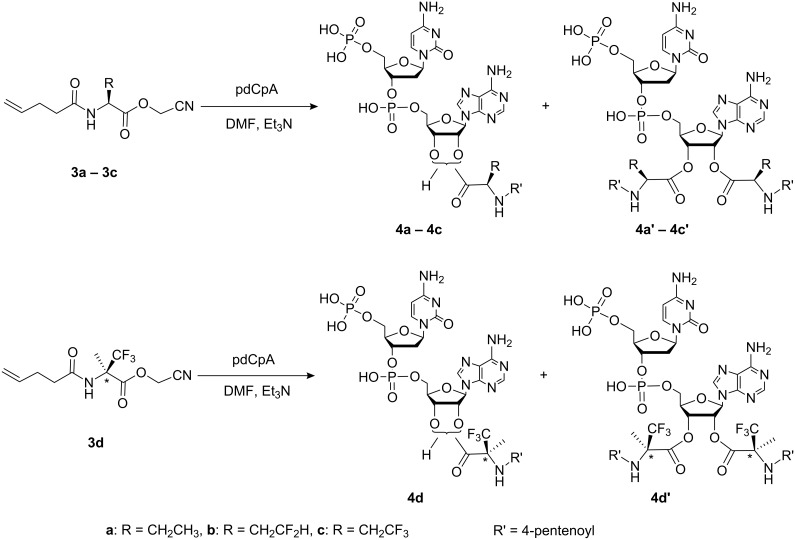
General scheme of synthesis of mono-2′(3′)-*O*-[*N*-(4-pentenoyl)aminoacyl]-pdCpAs and 2′-3′-bis-*O*-[*N*-(4-pentenoyl)aminoacyl]-pdCpAs.

**Figure 3:**
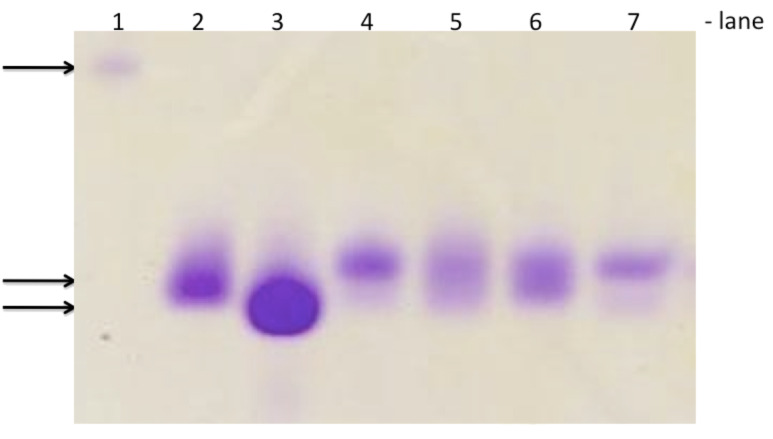
Denaturing PAGE of ligation products of truncated suppressor tRNA and fluorinated aminoacyl-pdCpAs and Abu-pdCpA. Lane 1: RNA Marker 100 bp, Lane 2: transcribed full-length tRNA_CUA_, Lane 3: transcribed truncated tRNA_CUA_-C_OH_, Lane 4: Abu-tRNA_CUA_, Lane 5: DfeGly-tRNA_CUA_, Lane 6: TfeGly-tRNA_CUA_, Lane 7: α-(Tfm)Ala-tRNA_CUA_. Amino groups are *N*-(4-pentenoyl) protected. Visualized by using Stains-all (Sigma-Aldrich^®^).

Chemical aminoacylation of pdCpA [4] was carried out using an N-protected amino acid activated as cyanomethyl ester in anhydrous DMF, and gave yields ranging from 40 to 90% (Scheme 2). The tetra-*n*-butylammonium (TBA) counter-ion is required to increase the solubility of pdCpA in DMF. Schultz and co-workers have reported that a ratio of 1:10 of TBA-pdCpA:activated ester results in the highly selective mono-acylation of the 2′,3′-hydroxyl groups of the adenosine ribose ring [5]. However, due to the expense of fluorinated amino acid analogues, we performed the aminoacylation reaction using a ratio of 1:2 or 1:3 TBA-pdCpA:activated ester at 40 °C overnight. Both mono-acylated and bis-acylated products were detected and purified by HPLC in the cases of Abu, TfeGly, and α-(Tfm)Ala, whereas only the mono-acylated product of DfeGly was observed. In general, longer incubation times and higher temperatures resulted in higher yields and increased amounts of bis-acylated products. A systematic investigation by Hecht and co-workers showed that such tandem activated tRNAs can also participate efficiently in the prokaryotic- and eukaryotic-based cell-free translation system. Both activated amino acids present in bis-acylated tRNAs can be recognized by the ribosome and incorporated into proteins [29–30]. pdCpA bearing Abu or the fluorinated amino acid analogue were efficiently ligated to the truncated suppressor tRNA^Phe^_CUA_ by treatment with T4-RNA ligase and were analyzed using denatured acidic PAGE (Figure 3) [31]. Thus, both our mono- and bis-acylated tRNAs are suitable for in vitro protein mutagenesis.

## Conclusion and Outlook

The efficient chemical and enzymatic synthesis of three novel fluorinated aminoacyl-pdCpAs and Abu-pdCpA and their corresponding charged tRNAs is reported. These aminoacyl-tRNAs can be used for site-specific protein mutagenesis in a cell-free protein synthesis system and will enable a systematic investigation of the structural and dynamic behavior of fluorine within a native protein environment.

## Supporting Information

Supporting information features detailed information on experimental procedures and compound characterization.

File 1 Experimental procedures and compound characterization
