# Supplementary material for: Chemical aminoacylation of tRNAs with fluorinated amino acids for in vitro protein mutagenesis
Source: Beilstein J Org Chem. 2010 Apr 20;6:40. doi: 10.3762/bjoc.6.40 (PMC2874332; doi:10.3762/bjoc.6.40)
Supplement: File 1 — Experimental procedures and compound characterization [file Beilstein_J_Org_Chem-06-40-s001.pdf]

Supporting Information

for

Chemical aminoacylation of tRNAs with fluorinated amino acids for in vitro protein mutagenesis

Shijie Ye, Allison Ann Berger, Dominique Petzold, Oliver Reimann, Benjamin Matt and Beate Koksch*

Address: Department of Biology, Chemistry, and Pharmacy, Freie Universität Berlin, Institute of Chemistry and Biochemistry – Organic Chemistry, Takustrasse 3, 14195 Berlin, Germany; Tel.: 0049-30-83855344; Fax: 0049-30-83855644.

E-mail: Beate Koksch - koksch@chemie.fu-berlin.de

*Corresponding author

Experimental procedures and compound characterization

Experimental

General

All reagents of synthetic grade were used as supplied. Anhydrous *N*,*N*-dimethylformamide (DMF), tetra-*n*-butylammonium hydroxide · 30 H_2O, *N*-4-pentenoyl chloride, tris (tetrabutylammonium)hydrogen pyrophosphate, and Stains-all were purchased from Sigma-Aldrich®. Iodoactonitrile and Dowex 50Wx8 ion exchange resin (20-50 mesh) were purchased from Fluka. (*S*)-2-Aminobutyric acid (Abu) was purchased from Bachem. (*S*)-2-Amino-4,4-difluorobutyric acid (DfeGly) [1], (*S*)-2-amino-4,4,4-trifluorobutyric acid (TfeGly) [2], (*RS*)-2-amino-2-methyl-3,3,3-trifluoropropanoic acid (TfmAla) [3], pdCpA [4], and 4-pentenoyloxy succinimide [5] were synthesized according to literature procedures. Acetonitrile (MeCN) for HPLC and 4-dimethylaminopyridine (DMAP) were purchased from Acros Organics. Deionized water for buffer solution was prepared using the MiliQ-Advantage A10-System (Millipore). T4 RNA ligase and restriction endonucleases *Fok*I, *BstN*I were purchased from New England Biolabs. Plasmid pYRNA8 encoding the yeast suppressor $tRNA^{Phe}_{CUA}$ was kindly provided by Prof. S. M. Hecht (Arizona State University , Tempe, AZ, USA).

Thin layer chromatography (TLC) for controlling the reaction process was performed on Merck silica gel 60 F254 plates. Flash column chromatography was carried out using Sigma Aldrich Kieselgel 60 silica gel (230–400 nm mesh). Analytical reverse-phase HPLC was performed on an Elite LaChrom HPLC (VWR & HITACHI) epuipped with an Organizer, an L-2200 Autosampler, two L-2130 pumps, and an L-2455 Diode Array Detector. A Capcell pak C18 column (Shisheido, Japan, Type: SG120 5μm, Size: 4.6 mmϕ x 250 mm) was used. Crude pdCpA and curde aminoacylated products were purified using C18 column (Phenomenex, USA, Type: Gemini-NX 10μ 110A AXIA, Size: 250 x 21.20 mm) on a reverse-phase HPLC (Knauer GmbH, Berlin, Germany) equipped with Smartline manager 5000 System, two smartline pumps 1000 and a UV detector 2500.

^{1}H, ^{13}C, and ^{19}F NMR spectra were obtained on Bruker ECX 400 MHz NMR spectrometer. Proton chemical shifts are reported in parts per million (ppm) and

referenced to the residual proton peak of $CDCl_3$. Spectral coupling patterns are reported as follows; b: broad s: singlet; d: doublet; t: triplet, and m: multiplet. Electrospray-ionization time-of-flight high resolution mass spectrometry (ESI-TOF) was performed on an Agilent 6210 ESI-TOF (Agilent Technologies, Santa Clara, CA, U.S.A.).

Synthesis of *N*-(4-pentenoyl)-(*S*)-2-aminobutyric acid cyanomethyl ester (3a)

A solution of 6 mL H_2O and 6 mL dioxane containing 226.4 mg Abu (2.2 mmol) and 357.2 mg $NaHCO_3$ (4.3 mmol) was treated with 465.1 mg *N*-4-pentenoyloxy succinimide (2.4 mmol) and stirred at room temperature for 17 h. The reaction mixture was diluted with 50 mL aqueous $NaHSO_4$ solution (1 M) and extracted three times with ethyl acetate (50 mL). The combined organic phase was dried with $MgSO_4$ and concentrated under reduced pressure. Compound **2a** was obtained as brown oil. The crude product was dissolved in 4 mL MeCN and treated with 1.5 mL triethylamine (10 mmol) and 0.72 mL iodoacetonitrile (10 mmol). After stirring at room temperature for 20 hours, the mixture was dissolved in 50 mL ethyl acetate and washed twice with 1 M aqueous $NaHSO_4$ solution. The organic layer was dried with $MgSO_4$ and concentrated under reduced pressure. The crude product was purified on a silica gel column (12 x 3 cm) with 4:1 ethyl acetate:hexanes. Compound **3a** was obtained as gray solid: yield 399.8 mg (81.25%); ^{1}H NMR (400 MHz, $CDCl_3$) δ: 0.94 (t, 3H, *J* = 7.4 Hz), 1.66–1.78 (m, 1H), 1.83-1.95 (m, 1H), 2.29–2.41 (m, 4H), 4.58 (td, 1H, *J* = 7.4 Hz, *J* = 5.4 Hz), 4.70 (d, 1H, *J* = 15.7 Hz), 4.82 (d, 1H, *J* = 15.7 Hz), 4.98-5.10 (m, 2H), 5.74-5.86 (m, 1H), 6.06 (bd, 1H, *J* = 7.0 Hz); ^{13}C NMR (400MHz, $CDCl_3$) δ: 9.74 ($-CH_3$), 25.30 ($-\underline{C}H_2CH_3$), 29.43 ($CH_2=CH_2-\underline{C}H_2-$), 35.45 ($-CH_2-\underline{C}H_2-$CO-), 48.90 ($-\underline{C}H_2-$CN), 53.07 (-NH-CH-), 114,05 (-CN), 115.92 ($\underline{C}H_2=$CH-), 136.81 ($CH_2=\underline{C}$H-), 171.26 (-CO-O-), 172.45 (-CO-NH-); mass spectrum (ESI-TOF), *m/z* 225.1263 $(M+H)^+$ ($C_{11}H_{16}N_2O_3$, calculated *m/z* 224.1161).

Synthesis of *N*-(4-pentenoyl)-(*S*)-2-amino-4,4-difluorobutyric acid cyanomethyl ester (3b)

60 mg DfeGly (0.43 mmol) was dissolved in a mixture of 1.5 mL water and 1.5 mL dioxane. To the mixture were added 72.3 mg $NaHCO_3$ (0.86 mmol) and 100 mg *N*-4-pentenoyloxy succinimide (0.51 mmol), and the reaction was stirred at room temperature for 18 h. The mixture was treated with 12 mL aqueous $NaHCO_3$ (1M) and extracted three times with 12 mL ethyl acetate. The combined organic phase was dried with $MgSO_4$ and concentrated under reduced pressure. The crude product was dissolved in 1.5 mL MeCN and treated with 0.35 mL triethylamine (2.5 mmol) and 0.16 mL iodoacetonitrile (2.2 mmol). After stirring at room temperature for 20 h, the reaction mixture was dissolved in 12 mL ethyl acetate and washed three times with 12 mL aqueous $NaHSO_4$ (1 M). The organic layer was dried ($MgSO_4$) and concentrated under reduced pressure. The crude product was purified on a silica gel column (20 x 1.5 cm) and eluted stepwise with 50 mL 1:2 ethyl acetate:hexanes and 50 mL 2:3 ethyl acetate:hexanes to give compound **3c** as a yellow oil: yield 66 mg (59%). ^{1}H NMR (400 MHz, $CDCl_3$) δ: 2.24-2.56 (m, 6H), 4.74 (d, 1H, *J* = 15.7 Hz), 4.80 (d, 1H, *J* = 15.7 Hz), 4.71–4.83 (m, 1H), 4.97–5.09 (m, 2H), 5.73–5.85 (m, 1H), 5.93 (tt, 1H, *J* = 55.8 Hz, *J* = 4.3 Hz), 6.59 (bd, 1H, *J* = 7.5 Hz); ^{13}C NMR (400 MHz, $CDCl_3$) δ: 29.26 (CH_2=CH-$\underline{C}H_2$-), 35.23 (-$\underline{C}H_2$-CO-), 35.63 (t, *J* = 22.0 Hz, $\underline{C}H_2$-CF_2H), 47.43 (t, *J* = 5.7 Hz, -NH-$\underline{C}$H-), 49.46 (-O-$\underline{C}H_2$-), 113.86 (-CH_2-$\underline{C}$N), 115.15 (t, *J* = 238.5 Hz, -CH_2-$\underline{C}F_2$H), 115.99 ($\underline{C}H_2$=CH-), 136.59 (CH_2=$\underline{C}$H-) 169.69 (-$\underline{C}$O-O-), 172.76 (-CO-NH-); ^{19}F NMR (400 MHz, $CDCl_3$) δ: -116.46 (tdd, *J* = 320.0 Hz, *J* = 58.6 Hz, *J* = 17.1 Hz), -115.70 (tdd, *J* = 320.0 Hz, *J* = 58.6 Hz, *J* = 17.1 Hz); mass spectrum (ESI-TOF), *m/z* 261.1045 $(M+H)^+$ ($C_{11}H_{14}F_2N_2O_3$, calculated *m/z* 260.0972).

Synthesis of *N*-(4-pentenoyl)-(*S*)-2-amino-4,4,4-trifluorobutyric acid cyanomethyl ester (3c)

60.1 mg $NaHCO_3$ (0.72 mmol) and 53.5 mg TfeGly (0.34 mmol) were dissolved in a mixture of 1.5 mL water and 1.5 mL dioxane, and 87.3 mg *N*-4-pentenoyloxy succinimide (0.44 mmol) added. After stirring for 19 h, the reaction mixture was diluted with 15 mL aqueous $NaHSO_4$ solution (1 M) and extracted three times with ethyl acetate (15 mL). The combined organic phase was dried using $MgSO_4$ and concentrated under reduced pressure. The crude product was dissolved in 1.5 mL

MeCN and 0.25 mL triethylamine (1.8 mmol), and 0.15 mL iodoacetonitrile (2 mmol) added. The resulting solution was stirred for 48 h at room temperature then diluted with 12 mL ethyl acetate and washed twice with 12 mL $NaHSO_4$ solution (1 M). The organic layer was dried ($MgSO_4$) and concentrated under vacuum. The crude product was purified on a silica gel column (13 x 2 cm) and eluted with 4:1 ethyl acetate:hexanes. Concentration under vacuum gave compound **3c** as a white solid: yield 71.2 mg (75.3%). ^{1}H NMR (400 MHz, $CDCl_3$) δ: 2.29–2.43 (m, 4H), 2.65–2.89 (m, 2H), 4.77 (d, 1H, *J* = 15.8 Hz), 4.83 (d, 1H, *J* = 15.8 Hz), 4.80–4.84 (m, 1H), 5.00–5.12 (m 2H), 5.74–5.88 (m, 1H), 6.32 (bd. 1H, *J* = 7.4 Hz); ^{13}C NMR (400 MHz, $CDCl_3$) δ: 29.16 (CH_2=CH-$\underline{C}H_2$-), 34.89 (-NH-$\underline{C}$H-), 35.03 (q, *J* = 28.0 Hz, -CH_2-$\underline{C}H_2$-CO-), 47.29 (q, *J* = 2.7 Hz, -$\underline{C}H_2$-CF_3), 49.63 (-O-$\underline{C}H_2$-), 113.52 (-CH_2-$\underline{C}$N), 116.10 ($\underline{C}H_2$=CH-), 125.50 (q, *J* = 276.0 Hz, -CH_2-$\underline{C}F_3$), 136.50 (CH_2=$\underline{C}$H-), 168.67 (-CO-O-), 172.44 (-CO-NH-); ^{19}F NMR (400 MHz, $CDCl_3$) δ: -62.98 (t, *J* = 9.8 Hz); mass spectrum (ESI-TOF), *m/z* 279.0929 $(M+H)^+$ ($C_{11}H_{13}F_3N_2O_3$, calculated 278.0878).

Synthesis of *N*-pentenoyl (*RS*)-2-amino-2-methyl-3,3,3-trifluoropropanoic acid cyanomethyl ester (3d)

A mixture of 361.8 mg TfmAla (2.3 mmol) and 10 mL DMF was treated with 0.5 mL 4-pentenoyl chloride (4.6 mmol) and 847.9 mg DMAP. After stirring at room temperature for 5 days, the mixture was diluted in 50 mL aqueous $NaHSO_4$ (1M) and extracted three times with ethyl acetate. The combined organic layer was dried with $MgSO_4$ and concentrated under reduced pressure to give 240.1 mg compound **2d** as a brown oil. The crude product was used in the next synthesis step without any further purification. 149 mg crude compound **2d** (ca. 0.6 mmol) was dissolved in 3 mL MeCN and treated with 0.25 mL triethylamine (1.8 mmol) and 0.2 mL iodoacetonitrile (2.7 mmol). The mixture was stirred at room temperature overnight. TLC showed incomplete disappearance of starting material, and a further quantity of iodoacetonitrile (0.2 mL) was added. After stirring for a total of 40 h, the reaction mixture was diluted in 25 mL ethyl acetate and washed twice with 25 mL aqueous $NaHSO_4$ (1 M). The organic layer was dried with $MgSO_4$ and concentrated under reduced pressure. The crude product was purified on a silica gel column (13 x 2 cm) and eluted with 1:1 ethyl acetate:hexanes. Concentration under vacuum gave

compound **3d** as a yellow oil: yield 87.4 mg (22%); ^{1}H NMR (400 MHz, $CDCl_3$) δ: 1.70 (s, 3H), 2.28–2.42 (m, 4H), 4.76 (d, 1H, *J* = 15.7 Hz), 4.83 (d, 1H, *J* = 15.7 Hz), 4.95–5.13 (m, 2H), 5.71–5.83 (m, 1H), 6.30 (s, H); ^{13}C NMR (400 MHz, $CDCl_3$) δ: 18.58 (-$\underline{C}H_3$), 29.10 (-$\underline{C}H_2$-CH_2-), 34.92 (-CH_2-$\underline{C}H_2$-), 49.87 (-$\underline{C}H_2$-CN), 61.23 (-NH-$\underline{C}$H-), 113.75 (-CH_2-$\underline{C}$N) 116.31 ($\underline{C}H_2$=CH-), 123.73 (q, *J* = 284.0 Hz, -CH_2-$\underline{C}F_3$), 136.31 (CH_2=$\underline{C}$H-), 165.29 (-CO-O-), 172.28 (-CO-NH-); ^{19}F NMR (400 MHz, $CDCl_3$) δ: -76.2267 (s); mass spectrum (ESI-TOF) *m/z* 279.0929 $(M+H)^+$ ($C_{11}H_{13}F_3N_2O_3$, calculated 278.0878).

Ion exchange chromatography of pdCpA

The TBA salt of pdCpA was prepared using Dowex 50Wx8, 20-50 mesh in its TBA form. The ion exchange beads (ca. 5 g) were washed with water, then stirred in 25 mL of 20% aqueous tetra-*n*-butylammonium for two hours. The beads were packed into a column and washed with water until the pH of the wash was neutral. An aqueous pdCpA-ammonium solution was passed over the column with water as the eluant. Fractions containing the TBA-pdCpA salt were determined by TLC and UV light of 254 nm wavelength. Additional tetra-*n*-butylammonium hydroxide was added to the solution. Lyophilzation yielded the tetra-*n*-butylammonium salt of pdCpA as a fluffy white powder. The product was dissolved in anhydrous DMF and stored in a sealed vial under argon at -20 °C (compound can be so stored for several weeks).

Synthesis of 2´(3´)-*O*-[*N*-(4-pentenoyl)-(*S*)-2-amino butyl]-pdCpA and bis-2´,3´-*O*-[*N*-(4-pentenoyl)-(*S*)-2-amino butyl]-pdCpA

To a flame-dried vial fitted with a magnetic stir bar and under an argon atmosphere was added 200 μl stock solution of the TBA salt of pdCpA in anhydrous DMF (25.5 μmol pdCpA). To this solution were added 14.4 mg compound **3a** (64.3 μmol) and 50 μL triethylamine. The reaction mixture was stirred at 40 °C overnight. A 5 μL aliquot of the reaction mixture was diluted with 45 μL 2:1 50 mM NH_4OAc:MeCN solution (pH 4.5) and analyzed by HPLC. Once the reaction was complete, the reaction mixture was diluted with 500 μL of 2:1 50mM NH_4OAc:MeCN solution (pH 4.5), loaded onto a reverse-phase C18 HPLC column and eluted with a gradient of

(10→80%) MeCN in 50 mM NH_4OAc buffer (pH 4.5) over a period of 40 min at a flow rate of 20 mL/min (monitoring at 260 nm). After lyophilization of the appropriate combined fractions, compound **4a** was obtained as colorless solid (retention time 7.4 min by preparative reverse-phase C18 HPLC column): yield 6.9 mg (33.7%), mass spectrum (ESI-TOF): *m/z* 804.2135 $(M+H)^+$ ($C_{28}H_{39}N_9O_{15}P_2$, calculated 803.2041). Compound **4a´** was obtained as colorless solid (retention time 13.1 min on preparative reverse-phase C18 HPLC column): yield 2.4 mg (9.6%), mass spectrum (ESI-TOF): *m/z* 971.2965 $(M+H)^+$ ($C_{37}H_{52}N_{10}O_{17}P_2$, calculated 970.2987).

Synthesis of 2´(3´)-*O*-[*N*-(4-pentenoyl)- (*S*)-2-amino-4,4-difluorobutyl]-pdCpA (4b)

To a flame-dried conical vial containing 12.6 mg compound **3b** (48.5 μmol) was added 10.8 mg pdCpA (16.98 μmol) as the TBA salt in 200 μL of DMF and 50 μL of triethylamine. The reaction mixture was stirred at 40 °C overnight. The reaction process was monitored by HPLC. Upon completion of the reaction, the mixture was diluted with 2:1 50 mM NH_4OAc:MeCN (pH 4.5) to a total volume of 500 μL and purified using reverse-phase HPLC. The compound was eluted with a gradient of 0→75% MeCN in 50 mM NH_4OAc over a period of 40 min at a flow rate of 20 mL/min (monitoring at 260 nm). After lyophilization of the appropriate combined fractions, compound **4b** was obtained as colorless solid (retention time 19 min on preparative reverse-phase C18 HPLC column): yield 9.3 mg (65%), mass spectrum (ESI-TOF): *m/z* 840.1857 $(M+H)^+$ ($C_{28}H_{37}F_2N_9O_{15}P_2$, calculated 839.1852).

Synthesis of 2´(3´)-*O*-[*N*-(4-pentenoyl)- (*S*)-2-amino-4,4,4-trifluorobutyl]-pdCpA (4c)

To a flame-dried conical vial containing 16.7 mg **3c** (60.1 μmol) was added 12.35 mg pdCpA (19.4 μmol) as the TBA salt in 200 μL of DMF and 50 μL of triethylamine. The reaction mixture was sirred at 40 °C overnight. The reaction was monitored by analytical reverse-phase HPLC. Upon completion, the reaction mixture was diluted with 2:1 50 mM NH_4OAc:MeCN (pH 4.5) to a total volume of 500 μL and purified by reverse phase HPLC. The compound was eluted with 10→80% MeCN in 50 mM

aqueous NH_4OAc over a period of 45 min at a flow rate of 20 mL/min (monitoring at 260 nm). After lyophilization of the appropriate combined fractions, compound **4c** was obtained as a white solid (retention time 9.5 min by preparative reverse-phase C18 HPLC column): yield 6.7 mg (13%), mass spectrum (ESI-TOF): *m/z* 858.1796 $(M+H)^+$ ($C_{28}H_{36}F_3N_9O_{15}P_2$, calculated 857.1758). Compoud **4c´** was also obtained as a white solid (retention time 13.1 min on preparative reverse-phase C18 HPLC column): yield 10.2 mg (48.7%), mass spectrum (ESI-TOF): *m/z* 1079.2612 $(M+H)^+$ ($C_{37}H_{46}F_6N_{10}O_{17}P_2$, calculated 1078.2421).

Synthesis of 2´(3´)-*O*-[*N*-(4-pentenoyl)-(*RS*)-2-amino-2-methyl-3,3,3-trifluoropropyl]-pdCpA (4d) and Bis-2´,3´-*O*-[*N*-(4-pentenoyl)-(*RS*)-2-amino-2-methyl-3,3,3-trifluoropropyl]-pdCpA (4d´)

To a flame-dried conical vial containing 16 mg compound **3d** (57.6 μmol) was added 20 mg pdCpA (31.4 μmol) as the TBA salt in 300 μL of DMF and 50 μL of triethylamine. The reaction mixture was stirred at 40 °C overnight. The reaction was monitored by analytical reverse phase HPLC. Upon completion, the reaction mixture was diluted with 2:1 50 mM NH_4OAc:MeCN (pH 4.5) to a total volume of 500 μL and purified using reverse phase HPLC. The compounds were eluted with a gradient of 0→75% MeCN in 50 mM aqueous NH_4OAc over a period of 40 min at a flow rate of 20 mL/min (monitoring at 260 nm). After lyophilization of the appropriate combined fractions, compound **4d** was obtained as a white solid (retention time 15.5 min on preparative reverse-phase C18 HPLC column): yield 12 mg (45%), mass spectrum (ESI-TOF): *m/z* 858.1796 $(M+H)^+$ ($C_{28}H_{36}F_3N_9O_{15}P_2$, calculated 857.1758). Compound **4d´** was obtained as a white solid (retention time 21.5 min on preparative reverse-phase C18 HPLC column): yield 15.4 mg (50.5%), mass spectrum (ESI-TOF): *m/z* 1079.2445 $(M+H)^+$ ($C_{37}H_{46}F_6N_{10}O_{17}P_2$, calculated 1078.2421).

In vitro transcription of truncated suppressor tRNA$^{Phe}_{CUA}$

Plasmid pYRNA8 encoding the yeast phenylalanine suppressor tRNA$^{Phe}_{CUA}$ was linearized with either the restriction endonuclease *Fok*I or restriction endonuclease *BstN*I (linearization with *Fok*I yields the –CA truncated tRNA and linearization with *BstN*I yields the full-length tRNA). Hecht reference then transcribed overnight using the AmpliScribe T7 RNA polymerase transcription kit at 42 °C. The transcription mixtures were first treated with RNase-free DNase I (included in the trancription kit) then extracted with phenol-chloroform. The tRNAs were pricipitated by adding 3M sodium acetate to a final concentration of 0.3 M and 2.5 volume equivalents of ice cold ethanol. The tRNAs were collected by centrifugation (4 °C, 13,000g, 10 min), washed with 250 μL 70% ethanol, and dried. The pellet was dissolved in nuclease-free water.

General Procedure: Synthesis of *N*-4-pentenoyl-aminoacyl-tRNAs

A mixture (final volume 50 μL) containing 40 nmol aminoacyl-pdCpA, 10 μg truncated tRNA (0.4 nmol), 15 mM $MgCl_2$, 1 mM ATP, 50 ng/μL BSA, 10% DMSO (v/v), 50 mM Heps buffer (pH 7.5) and 100 units of T4-RNA ligase was incubated at 37 °C for 45 min. The reaction was quenched by adding 5 μl of 3M NaOAc solution (pH 5.2) and extracted with phenol-chloroform. *N*-4-pentenoyl-aminoacyl-tRNA was precipitated by adding aqueous NaOAc solution (pH 5.2) to a final concentration of 0.3 M and 2.5 volume equivalents of ice-cold ethanol. *N*-4-pentenoyl-aminoacyl-tRNA was colleted by centrifugation (4 °C, 13,000g, 10 min). The pellet was washed with 250 μL 70% ethanol, dried, and dissolved in 0.1 M aqueous sodium acetate solution (pH 5.2).

General Procedure: Acid urea polyacrylamide gel electrophoresis

The ligation efficiency was analyzed by an acid urea PAGE (20 x 20 cm) [6]. A tRNA solution was mixed with equal volumes of 2x RNA loading buffer give recipe, denatured by heating at 70 °C for ten minutes and chilling on ice. Samples were loaded onto a 1-mm-thick 7% gel (acrylamide:bisacrylamide 19:1) containing 8M urea in 0.1 M sodium acetate buffer (pH 5.2). Electrophoresis was performed at 200 V

(10 V/cm) for 3.5 h, until the bromphenol blue dye band reached the bottom of the gel. Gels were stained with Stains-All (Sigma-Aldrich).

References

1. Tsushima, T.; Kawada, K.; Ishihara, S.; Uchida, N.; Shiratori, O.; Higaki, J.; Hirata, M. *Tetrahedron* **1988,** *44*, 5375–5387. doi:10.1016/S0040-4020(01)86044-0

2. Winkler, D.; Burger K. *Synthesis* **1996,** 1419–1421. doi:10.1055/s-1996-4409

3. Smits, R.; Cadicamo, C. D.; Burger, K. and Koksch, B. *Chem. Soc. Rev.* **2008,** *37,* 1727–1739. doi:10.1039/b800310f

4. Robertson, S. A.; Noren, C. J.; Anthony-Cahill, S. J.; Griffith, M. C.; Schultz, P. G. *Nucleic Acids Res.* **1989,** *17,* 9649–9660. doi:10.1093/nar/17.23.9649

5. Lodder, M.; Wang, B. X.; Hecht, S. M. *Methods* **2005**, *36*, 245–251. doi:10.1016/j.ymeth.2005.04.002

6. Köhrer, C.; Rajbhandary, U. L. *Methods* **2008,** *44,* 129–138. doi:10.1016/j.ymeth.2007.10.006
